# Clinic of the Jefferson Medical College

**Published:** 1851-04

**Authors:** G. R. Morehouse

**Affiliations:** Senior Clerk of the Surgical Department


*Clinic of the Jefferson Medical College. Service of Professors* PANCOAST *and* MÜTTER. Reported by G. R. MOREHOUSE, M. D., senior Clerk of the Surgical Department.

*Clinic of January 4th,* 1851.

Cases presented this day:—Ophthalmia tarsi, of sixteen years duration; Bronchocele; Thecal abscess; Dislocation of Radius, *backwards;* Caries of Turbinated bones; Inversion of Toe-nail; Perforating ulcer of the Cornea; Spasmodic stricture of Œsophagus; Congenital fissure of the Soft Palate; Urinary Calculus; Scrofulous Eruption of Skin; Pterygium; Ozœna; Syphilitic Ulceration of the Arm, extensive; Cataract; Varicose enlargement of the Crural Veins; Amaurosis; Hydrocele; Scrotal Hernia; Irritable Ulcer of the Leg; Enlarged Tonsils; Cancer of Breast; Ulceration of Soft Palate; Psoas Abscesses; Lipoma Diffusum; Albugo; Strabismus convergens double; Strabismus Divergens; Ulceration of the Thigh; Spastic contraction of Pectineus muscle; Fistula Lachrymalis; with caries of Os Unguis; Prolapsus Iridis; Caries of head of Femur; Conjunctivitis; Laceration of External Lateral Ligaments of Ankle; Scrofulous Tumor of Neck, very large; Fracture of Fibula; Tumor of the Penis, analogous to a Bubo; Cancer of Pylorus; Injury of Lumbar Plexus; Lepra Syphilitica; Otorrhœa. Total 44.

The following cases were operated upon before the class, by Prof. Pancoast.

*Case* 1*st, Pterygium Musculosum.*

The patient, Thomas Johnson, æt. 24, has for some time past, been laboring under chronic inflammation of the conjunctiva; this however, has subsided, leaving apparently, as a sequent, the disease which you here see. Upon examining the eye, we find a circumscribed, sub-conjunctival cellular structure, thick, elevated and highly vascular, of a triangular form, having its base incorporated with the plica semilunaris, and its apex extending towards the central point of the cornea. In color, this variety of Pterygium closely resembles muscular fibre, hence the name crassum or musculosum, to distinguish it from the Pterygium tenue which is thin, membranous, and semi-transparent. A Pterygium is not painful, and when confined to the sclerotic conjunctiva, gives little inconvenience; its removal, therefore, is seldom desired, until by encroaching upon the cornea, it forms an impediment to vision.

The plan of operation adopted by Prof. Pancoast, which is novel and very successful, is as follows: The patient is seated on a chair, and directed to turn the affected eye *out*, or *in*, as far as possible, according as the Pterygium is situated on the nasal, as it most usually is, or temporal side of the orbit. An assistant supports the head against his breast and separates the eye-lids as in the operation for cataract, or with the spring speculum employed by Dr. P. in Strabismus. The surgeon standing in front, then seizes the Pterygium with a pair of hooked forceps, raises it from the sclerotica and passes a delicate needle armed with the finest silk ligature beneath it, avoiding any encroachment upon the semi-lunar fold. The ligature is then tied, and that portion of the Pterygium on its corneal side removed with a delicate scalpel or curved pair of scissors, as in the old operation. In a few days the ligature comes away, the vessels are effectually sealed, and the risk of their reproduction in a great measure precluded. The cicatrisation of the raw surface left, is aided by slightly touching it from day to day with the caustic pencil.

*Case* 2d. *Enlargement of the Tonsils.*

John F. Scott, æt. 45, complains of a sense of fulness or obstruction about the throat, which is rendered especially evident during deglutition. His voice during eight years past has undergone marked alteration, becoming rough and thick; he is, moreover, subject to a continual hacking cough, with occasional inclination to vomit. Upon the slightest exposure all these symptoms become aggravated; sometimes his hearing is materially affected, and his articulation rendered indistinct. Upon examination, the tissue of the uvula and soft palate was found œdematous; the tonsils were enlarged to such an extent as almost to close the opening of the fauces; were possessed of little sensibility; feeling firm and resisting to the touch, and presented on their surfaces numerous ragged follicular orifices filled with tenacious mucus.

This chronic state of enlargement to which the tonsil is subject is generally the result of repeated attacks of acute inflammation, and is frequently found associated with the strumous diathesis.

The proximate cause is the hypertrophy of the areolar tissue in which the mucous follicles of the gland are imbedded; the repeated deposits of lymph which take place at each successive inflammation remaining unabsorbed. Enlarged tonsils are seldom painful, but are generally removed to obtain relief from the inconvenience their bulk occasions. When large they offer serious impediment to deglutition and phonation, and frequently produce deafness by blocking up the openings of the Eustachian tubes.

When the enlargement is chronic, local applications will prove of little avail, and partial extirpation becomes by far the most speedy and effectual treatment. To accomplish this, various methods have been proposed; the ligature and caustic have each met with strong advocates among the older surgeons, but excision in modern hands has proved most satisfactory, and at the same time most certain. If excision be decided upon, the operation may be thus performed. The gland being seized with a pair of hooked forceps or a tenaculum, is drawn tense, a probe pointed bistoury well wrapped at its base, to prevent the mouth being cut, should then be introduced beneath the gland, and a portion of the tumor removed, cutting upwards in a line parallel with the arches of the palate. The objection to this operation is, that in children and those in whom the fauces are irritable, the grasp of the forceps occasions such severe coughing as to render the use of the bistoury troublesome and dangerous. It is therefore advisable to employ for their removal the guillotine instrument of Dr. Physick, or some of its modifications. The oval instrument of Fahnestock with a spring forceps attached for the purpose of drawing the gland well within the grasp of the knife, is preferred by Prof. Pancoast. Not being capable of receiving a very keen edge, its use is thereby less liable to be followed by hæmorrhage. In the present case it was used successfully. The glands were removed and the patient ordered to use for a few days, an infusion of white oak bark as a gargle.

*Case 3d. Lipoma Diffusum.*

Alexander Matheson æt. 24, presents himself with a tumor over the right scapula: it is not painful, but its rapid enlargement combined with the inconvenience and deformity it occasions, has induced him to apply for its removal. The tumor presents all the external characteristics of that variety of fatty tumor designated Lipoma Diffusum; it is lobulated, freely movable, gives no pain upon handling, feels soft and slightly elastic to the touch, and has no well defined edges, but rises gradually from the surrounding tissue. The causes of fatty tumors are not known: they are frequently congenital and when occurring in adults can seldom be traced as the result of external injury. They depend upon an unnatural accumulation of fat in the enlarged fat cells, and are most frequently found upon the back and neck, presenting two varieties; those having a broad base, as in the case before you, and those attached by a neck or peduncle and generally of an oblong shape. Fatty tumors are generally seated in the superficial fascia; sometimes, however, they occupy the deeper areolar tissues, developing themselves under muscles and in other situations which render them more difficult of recognition. As an example of this, there was during the present winter a child between three and four years old presented at the clinic, with a very large congenital tumor, occupying the scrotum and extending up the chord and through the external abdominal ring, presenting very much the appearance of a large scrotal hernia. This, Dr. Pancoast observed, was in all probability, a local accumulation of fat, which some of the older surgeons have called, though improperly, adipose hernia. Upon removal, the tumor was found to be adipose in its character, having its origin from the adipose and areolar tissues of the spermatic cord within the inguinal canal.

When fatty tumors are small, their absorption sometimes, though rarely, may be effected by friction and iodine applications and pressure; the knife, however, supplies us with the only sure method of removal. The operation is seldom bloody or dangerous, but may be rendered so from the great size of the tumor, or its intimate relation with important parts, or the too free use of the knife. The incision through the integuments should be free, the cyst, when they are provided with one, fairly opened, and the tumor dexterously worked out with the forceps and the handle of the knife, the strong adhesions alone being divided with the edge. In the present case, the tumor was grasped about its middle, and raised from the tissues beneath; a curved bistoury was then plunged through its base, and the tumor and integument divided at one cut. Each half of the tumor was turned out from the healthy tissue enveloping it, and two small vessels tied. When all oozing had ceased, the wound was closed with stitches and adhesive plaster. The dressing consisted of lint spread with simple cerate, and a compress and roller to keep the surfaces of the wound in close apposition. The arm was put in a sling to keep the shoulders at rest.

*Case 4th. Cancer of Breast.*

The patient thus gives the history of the case. Sixteen years ago, her attention was directed to the left breast, by the appearance of a small moveable tumor, in the substance of the gland about an inch external to the nipple. As the disease advanced, the tissue surrounding the areola became indurated, and the nipple commenced receding, becoming thick and short and causing the skin by which it was covered to arrange itself in circular folds like a turban. At the expiration of six years, the nipple had entirely disappeared; the indurations embraced a more extensive surface, and the gland was subject to occasional attacks of pain.

The breast maintained this condition unaltered until the beginning of the past year; since then the paroxysms of pain have been more frequent, and lancinating; not being confined to the breast, but extending into the axilla and through to the back.

Upon examination, the integuments were found unbroken, although somewhat adherent to the hardened gland beneath; the nipple was entirely inverted, its site presenting a deep depression with an almost cartilaginous edge. The axillary glands were not sensibly affected. There was a constant feeling of uneasiness in the breast, which shortly after handling became converted into excruciating pain.

The removal of the breast was decided to be necessary. For this purpose the patient was placed in a chair, the arm held at right-angles from the body, and the skin made tense by an assistant. The surgeon sitting in front made an incision commencing at the anterior margin of the axilla, and carrying it below the nipple to the inner inferior boundary of the gland, cutting through the integument and superficial fascia. The second incision extended between the same points, being carried above the nipple. The gland was then dissected out, cutting from below upwards, and removed, with a portion of the pectoralis major muscle, to which it had become attached. The vessels were tied, and the wound left exposed to the air until oozing had ceased. The edges were then drawn together and retained by suture and adhesive plaster. The dressing consisted of lint spread with simple cerate, a compress and breast bandage drawn firmly over the wound. After the operation, she took a sedative mixture to prevent excessive febrile reaction.

*Case 5th. Prolapsus Iridis.*

Mary Ann Wiley, æt. 11, a fleshy child of strumous habit, has for some time been laboring under an attack of scrofulous corneitis. While playing with a fork she struck the cornea of the right eye with one of the prongs. The prong penetrated it at a distance of two lines from its junction with the sclerotic. A portion of the aqueous humour escaped, and subsequently the iris protruded to the degree designated myocephalon.

Professor Pancoast first endeavored to reduce the protrusion with a probe, and by gentle friction on the ball, but finding this impracticable, be snipped it off with a pair of scissors, and permitted the iris to retract. The child was put under the following treatment:

| | | |
|---|---|---|
| ℞. | Hydg. chld. Mit. | gr. $\frac{1}{3}$ |
| | Pulv. opii | $\frac{1}{6}$ |
| | Ext. Belladonnæ | $\frac{1}{6}$ |

To be taken twice daily until all the pain has ceased or the gums are touched. One-half a drachm of a mixture of equal parts of extract bellad. and mercurial ointment to be rubbed night and morning round the orbit. Both eyes to be covered with a soft compress and bandage, for the purpose of excluding the light, and keeping the lids at rest. The prognosis, after a puncture of the cornea with a blunt instrument, as in this case, especially if the iris or ciliary body be touched, is, in a majority of instances, unfavorable, so far as regards the preservation of sight. The loss of vision may arise from the inflammation of the lining membrane of the aqueous chamber and the iris, by which the pupil becomes permanently closed, or the iris fastened by a deposit of lymph, to the inner surface of the cornea (*synecha anterior*) or to the capsule of the lens, (*synecha posterior;*) or if the injury of these parts, be subdued by the treatment, blindness may follow in consequence of the lens, or its capsule, becoming opaque a few days subsequent to the accident. In either of these cases relief, should it become necessary in consequence of an accident to the other eye, may be obtained by a surgical operation, the formation of an artificial pupil, or the operation for cataract. But if the ciliary processes and choroid tunic suffer, another train of symptoms ensue, more especially to be dreaded, as it is very commonly followed not only by permanent loss of vision, but by great enlargement and deformity of the eye-ball. The inflammation of these vascular structures is accompanied with great pain and distension of the ball, and a profuse secretion of the aqueous humor. Finally the sclerotic coat yields to the distension, and lets the swollen veins of the ciliary processes, which have become large and varicose, rise up in the form of dark blue tumors round the base of the cornea. This becomes a hideous deformity, and the eye protrudes so as to form a case of exophthalmus. If the tumors be tapped to reduce the swelling, or a spontaneous diminution of the ball takes place subsequently, the ball is liable to shrink below its natural dimensions and become atrophied. The lecturer then enforced strongly upon the class, the importance of making a guarded prognosis, after wounds made through the front of the eye with blunt instruments, and endeavoring to check as speedily as possible by an antiphlogistic course of treatment, and by the use mercury, opium, and belladonna, the traumatic inflammation which may lead to such deplorable results. In the present case, he observed, in consequence of its complication with scrofulous corneitis, we may be compelled, in a day or two, to modify the plan of treatment by applying lunar caustic in substance to the corneal wound, or in solution to the conjunctiva, and giving quinia internally.

Note.—The results in the above cases have been most satisfactory. In the first case scarce any sign indicative of the previous existence of a Pterygium can be detected. The incisions in cases 3 and 4 healed rapidly. Both patients were discharged in three weeks, to all appearance cured. In case 5th, inflammation was kept in abeyance; the wound has healed and the eye is perfect, with the exception of slight cloudiness of the cornea.

